# Perspective on Dentoalveolar Manifestations Resulting From PHOSPHO1 Loss-of-Function: A Form of Pseudohypophosphatasia?

**DOI:** 10.3389/fdmed.2022.826387

**Published:** 2022-02-03

**Authors:** Fatma F. Mohamed, Michael B. Chavez, Flavia Amadeu de Oliveira, Sonoko Narisawa, Colin Farquharson, José Luis Millán, Brian L. Foster

**Affiliations:** 1 Division of Biosciences, College of Dentistry, The Ohio State University, Columbus, OH, United States,; 2 Sanford Children’s Health Research Center, Sanford Burnham Prebys Medical Discovery Institute, La Jolla, CA, United States,; 3 The Royal (Dick) School of Veterinary Studies (RDSVS), The Roslin Institute, University of Edinburgh, Edinburgh, United Kingdom

**Keywords:** hypophosphatasia, mineralization, dental, periodontal, genetic models

## Abstract

Mineralization of the skeleton occurs by several physicochemical and biochemical processes and mechanisms that facilitate the deposition of hydroxyapatite (HA) in specific areas of the extracellular matrix (ECM). Two key phosphatases, phosphatase, orphan 1 (PHOSPHO1) and tissue-non-specific alkaline phosphatase (TNAP), play complementary roles in the mineralization process. The actions of PHOSPHO1 on phosphocholine and phosphoethanolamine in matrix vesicles (MVs) produce inorganic phosphate (P_i_) for the initiation of HA mineral formation within MVs. TNAP hydrolyzes adenosine triphosphate (ATP) and the mineralization inhibitor, inorganic pyrophosphate (PP_i_), to generate P_i_ that is incorporated into MVs. Genetic mutations in the *ALPL* gene-encoding TNAP lead to hypophosphatasia (HPP), characterized by low circulating TNAP levels (ALP), rickets in children and/or osteomalacia in adults, and a spectrum of dentoalveolar defects, the most prevalent being lack of acellular cementum leading to premature tooth loss. Given that the skeletal manifestations of genetic ablation of the *Phospho1* gene in mice resemble many of the manifestations of HPP, we propose that *Phospho1* gene mutations may underlie some cases of “pseudo-HPP” where ALP may be normal to subnormal, but *ALPL* mutation(s) have not been identified. The goal of this perspective article is to compare and contrast the loss-of-function effects of TNAP and PHOSPHO1 on the dentoalveolar complex to predict the likely dental phenotype in humans that may result from *PHOSPHO1* mutations. Potential cases of pseudo-HPP associated with *PHOSPHO1* mutations may resist diagnosis, and the dental manifestations could be a key criterion for consideration.

## INTRODUCTION

Mineralization of skeleton occurs by several physicochemical and biochemical processes and mechanisms that facilitate the deposition of hydroxyapatite (HA) in specific areas of the extracellular matrix (ECM). Experimental evidence has pointed to the presence of HA crystals inside, and outside, collagen fibrils in the ECM (1, 2), and also within the lumen of matrix vesicles (MVs) (3–5). Production of inorganic phosphate (P_i_) for the initiation of HA mineral formation within the lumen of MVs takes place by the action of phosphatase, orphan 1 (PHOSPHO1) on phosphocholine and phosphoethanolamine in the inner leaflet of the MV membrane (6, 7). On the outer surface of the MV membrane, the glycosylphosphatidylinositol (GPI)-anchored tissue-non-specific alkaline phosphatase (TNAP) isozyme hydrolyzes both adenosine triphosphate (ATP) and mineralization inhibitor inorganic pyrophosphate (PP_i_) to generate P_i_ that is incorporated into MVs by the action of phosphate transporter 1 (P_i_T-1) (6, 8). These backup mechanisms explain why MVs isolated from chondro-osteogenic cells deficient in either TNAP or PHOSPHO1 retain the ability to initiate mineralization, although at reduced levels (9–11).

Genetic mutations in the *ALPL* gene-encoding TNAP lead to hypophosphatasia (HPP; OMIM# 241500, 241510, and 146300) characterized by low circulating alkaline phosphatase (ALP), rickets in children and/or osteomalacia in adults, and with a very broad range of severity with several clinical types (from most to least severe: perinatal lethal, infantile, severe or mild childhood, adult, and odontohypophosphatasia) (12). Those with the gravest forms of HPP (perinatal and infantile types) often die *in utero* or soon after birth because of severe skeletal hypomineralization, respiratory failure due to thoracic cage dysplasia and hypoplastic lungs, and elevated intracranial pressure due to craniosynostosis (13–16). Dentoalveolar phenotypes (described in detail below), particularly premature exfoliation of primary teeth, are commonly observed in patients with mild and severe subtypes of HPP (17–19). *Alpl* knockout (*Alpl*^−/−^) mice phenocopy infantile HPP extremely well, including their failure to thrive, high-pitched cries, rickets and extensive hypomineralization of their skeleton, and severe seizures that precede death within a few weeks after birth (though a pyridoxal-enforced diet can reduce seizures and slightly extend lifespan) (20–22). The term pseudo-HPP has been used to describe apparent cases of HPP where ALP levels are within normal range and may also apply when no genetic variant in *ALPL* can be identified (23–28).

Mice with a *Phospho1* gene ablation (*Phospho1*^−/−^) live to adulthood but develop scoliosis starting from birth, osteomalacia, greenstick fractures, accompanied by the elevation in plasma PP_i_ and a decrease in plasma TNAP activity (11, 29). Less pronounced and distinct dentoalveolar abnormalities (described in detail below) accompany PHOSPHO1 loss-of-function (29). Importantly, the simultaneous ablation of both the *Alpl* and *Phospho1* genes leads to embryonic lethality with a complete absence of skeletal and dental mineralization (11).

To our knowledge, no pathogenic, loss-of-function mutations in *PHOSPHO1* have been reported in humans. Given that the symptomatology of *Phospho1*^−/−^ mice (osteomalacia, spontaneous fractures, scoliosis) resembles many of the manifestations of HPP, we propose that a subset of individuals with HPP-like manifestations that resist diagnosis (i.e., some in the pseudo-HPP group) could potentially carry unreported pathogenic *PHOSPHO1* variants. The goal of this perspective article is to compare and contrast the loss-of-function effects of key mineralization-associated phosphatases, TNAP and *PHOSPHO1*, on the dentoalveolar complex to predict the dental phenotype in humans that may result from *PHOSPHO1* mutations. Potential cases of pseudo-HPP associated with *PHOSPHO1* mutations may resist diagnosis, and dental manifestations could be a key criterion for consideration.

## DENTAL MINERALIZATION DEFECTS ASSOCIATED WITH HYPOPHOSPHATASIA

An array of dental manifestations can accompany HPP, including enamel defects, thin and/or hypomineralized dentin, wide pulp chambers, root size and shape abnormalities, acellular cementum hypoplasia, alveolar bone loss, periodontal disease, tooth mobility, and malocclusion (17, 18, 30–32). Dental defects are variable across clinical HPP subtypes, though the premature loss of primary teeth is a hallmark of all types of HPP and can be a key diagnostic criterion, as teeth are exfoliated “fully rooted” (i.e., lacking substantial root resorption that normally marks primary tooth loss) spontaneously or resulting from minor traumas. Tooth loss is the result of acellular cementum hypoplasia and loss of periodontal attachment. As with other clinical manifestations of HPP, genotype –phenotype correlations are not well-understood for dental aspects of the disease. The mildest clinical form of HPP referred to as odontohypophosphatasia (odonto-HPP), features primarily tooth loss and other dental disorders in combination with low ALP but in the absence of other clinically evident signs.

**FIGURE 1 |:**
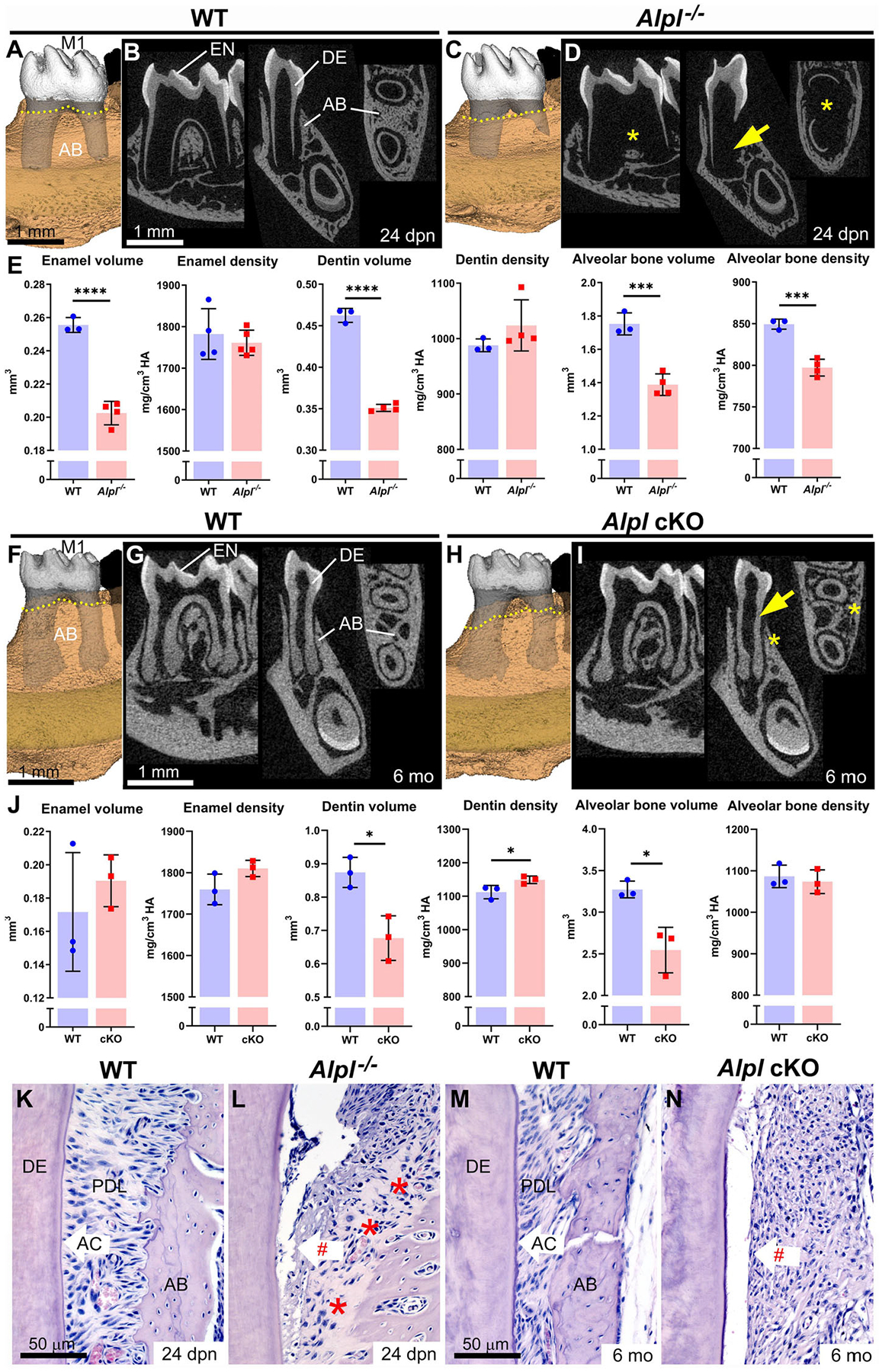
Dentoalveolar defects in mouse models of hypophosphatasia. **(A–D)** 3D and 2D micro-CT renderings of first molars (M1) reveal severe dentin (DE) defects (yellow arrows) and alveolar bone (AB) defects (yellow *) in *Alpl*^−/−^ vs. wild-type (WT) mice at 24 days postnatal (dpn). AB levels (yellow dotted lines in A and C) are already reduced in *Alpl*^−/−^ vs. WT mice. **(E)** Compared to WT, *Alpl*^−/−^ have reduced enamel (EN) volume, DE volume, and AB volume and density **(F–I)** 3D and 2D micro-CT renderings of M1 reveal thin DE (yellow arrow) and AB loss (yellow *) in *Alpl* cKO vs. WT mice. AB levels (yellow dotted lines in F and H) have declined significantly in *Alpl* cKO vs. WT mice by 6 months (mo). **(J)** Compared to WT, *Alpl* cKO has reduced DE and AB volumes. **(K–N)** Histology confirms that both *Alpl*^−/−^ and *Alpl* cKO mouse models lack acellular cementum (AC), resulting in periodontal ligament (PDL) detachment (red #). *Alpl*^−/−^ mice feature large areas of alveolar bone osteoid (red stars in panel L). Detailed analytical methods are included in the original publications, and as described in Chavez et al., JBMR Plus 5(3): e10474, 2021. Briefly, micro-CT generated DICOM images were analyzed with AnalyzePro 1.0 (AnalyzeDirect, Overland Park, KS), calibrated to five known densities of HA (mg/cm^3^ HA), and density thresholds were defined as >1,600 mg/cm^3^ HA for enamel and 450–1,600 mg/cm^3^ HA for dentin and bone. Statistical analyses included independent samples *t*-tests where graphs show means ± SD and results are indicated by: *p< 0.05; **p < 0.01; ***p < 0.001; ****P < 0.0001. **(A–E)** are republished with permission from Kramer et al., *Bone* 143:115732, 2021. The remaining images are from expanded, previously unpublished analyses from data presented in Foster et al. (34) and presented with permission.

*Alpl*^−/−^ mice phenocopy severe infantile HPP with <1% of circulating ALP activity compared to control mice, reflecting the range of severe dental defects observed in individuals with HPP, and serving as an important model to study disease mechanisms (20, 21, 31, 33). Compared to wild-type (WT) controls, *Alpl*^−/−^ mice show severe enamel and dentin hypoplasia and reduced alveolar bone volume and mineral density (Figures 1A–E) (30, 35–39). Some local defects in *Alpl*^−/−^ dentin and alveolar bone are so severe that whole regions of these tissues fall below the threshold for the detection by micro-CT, appearing invisible in 3D and 2D renderings (yellow arrows and stars in Figure 1D), and contributing to reduced volumes in affected tissues (35). Alveolar bone levels are already decreased in *Alpl*^−/−^ mice by 24 days postnatal (dpn), shortly after first molar eruption (yellow dotted lines showing alveolar crest height in Figures 1A vs. 1C). All mineralizing dentoalveolar cells express TNAP, including ameloblasts, odontoblasts, cementoblasts, and osteoblasts (30, 37, 40). Studies analyzing mechanisms for enamel defects indicate disruptions in ameloblast organization and enamel ultrastructure (37), while dentin defects arise from inhibition of mineralization outside of MVs in the dentin matrix (30). These dentin defects initiate in the outer mantle dentin layer where MVs are critical for the initiation of mineralization, and in more severe cases, dentin hypomineralization also spreads to circumpulpal dentin, as indicated in mouse, sheep, and human studies of HPP (30, 35, 41).

*Alpl*^−/−^ mice have lethality within the first few weeks after birth, precluding their use in longer-term studies on pathology and treatment effects. A mouse that harbored a floxed *Alpl* allele was designed to produce tissue-specific conditional deletion of *Alpl* when crossed with Cre recombinase-expressing mouse lines, allowing more refined deletion and less severe and/or later onset of disease. *Col1a1*-Cre-mediated deletion of *Alpl* in conditional knockout (cKO) mice affected reduced circulating ALP levels by about 75% and mice lived to the termination of the study at 6 months (34). These cKO mice resembled childhood-onset HPP, with axial and appendicular skeletal defects, long bone fractures, and defects in dentin and alveolar bone that became apparent prior to adulthood (Figures 1F–J).

While enamel and dentin defects in mouse models of HPP are well-represented in reports on developmental phenotypes, the longer lifespan of cKO vs. *Alpl*^−/−^ mice allowed assessment of tissues later in life, leading to the first observation of severe periodontal bone loss in a mouse HPP model (yellow dotted lines showing alveolar crest height in Figures 1F vs. 1H). Histology reveals that both *Alpl*^−/−^ and cKO mouse models of HPP have prominent acellular cementum hypoplasia, resulting in the poor periodontal attachment (Figures 1K–N). This defect is considered the primary driver of primary and secondary tooth loss in individuals with HPP and contributes to periodontal disease, mobility, and malocclusion.

In summary, while effects of HPP are highly variable, they can potentially impact all dentoalveolar mineralized tissues, in part because TNAP is highly expressed by all mineralizing cells during dental development and tissue mineralization. A mouse model that harbored a knock-in *Alpl* A116T substitution mutation associated with odonto-HPP (42) showed about 50% decreased circulating ALP levels and mild effects on acellular cementum and alveolar bone, possibly the two most sensitive tissues (33).

## DENTAL EFFECTS OF PHOSPHO1 LOSS-OF-FUNCTION IN MICE

**FIGURE 2 |:**
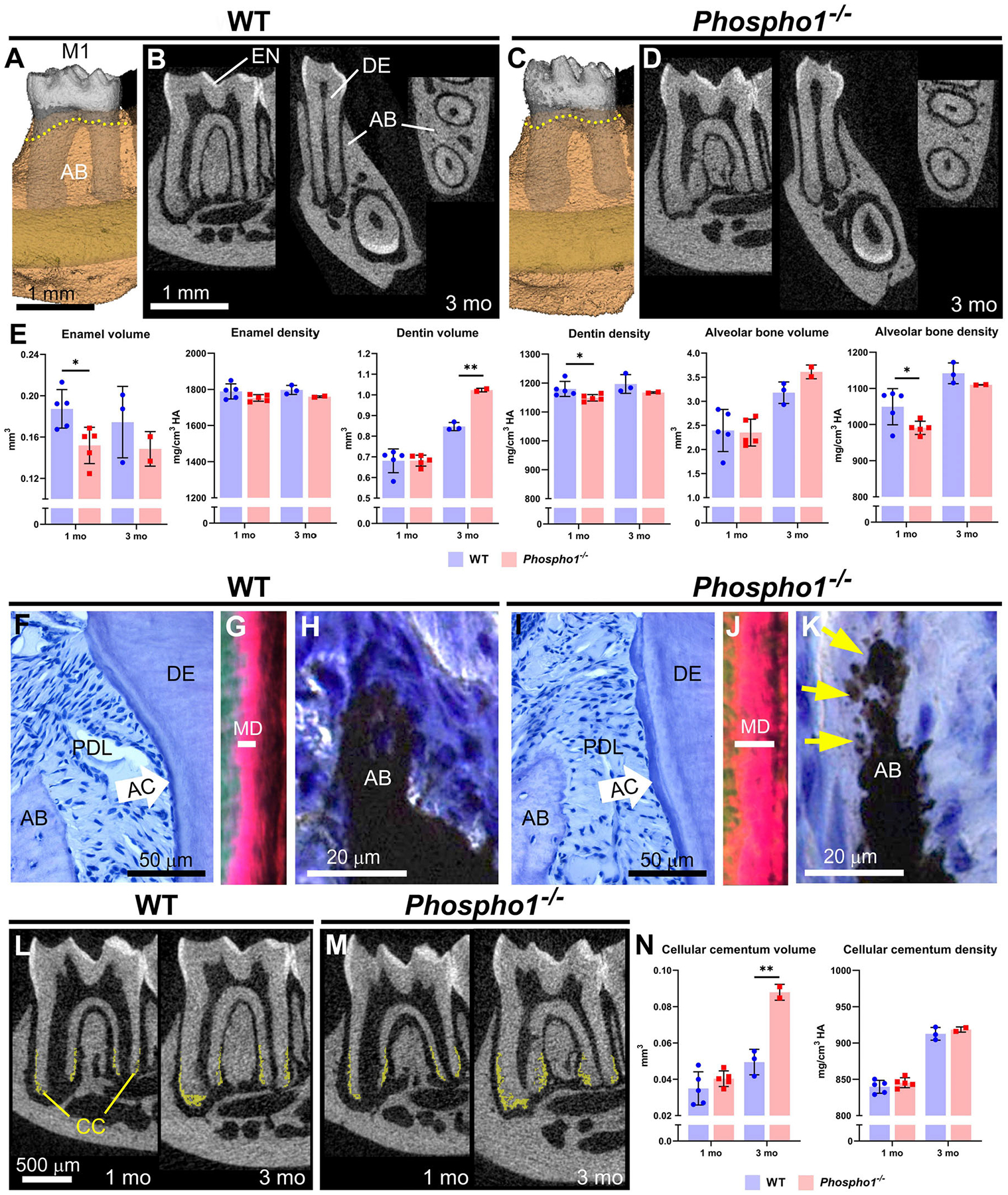
Dentoalveolar defects in *Phospho1*^−/−^ mice. **(A–D)** 3D and 2D renderings of first molars (M1) from WT and *Phospho1*^−/−^ mice at 3 months (mo), including enamel (EN), dentin (DE), and alveolar bone (AB). AB levels (yellow dotted lines in A and C) are similar in *Phospho1*^−/−^ vs. WT mice at 3 mo. **(E)** Compared to wild-type (WT), *Phospho1*^−/−^ mice have mild enamel hypoplasia and reduced DE and AB mineral densities at 3 mo. **(F, I)** H&E stain of WT and *Phospho1*^−/−^ dentoalveolar tissues at 3 mo showing intact acellular cementum (AC) layer and periodontal ligament (PDL) attachment. **(G, J)** Picrosirius red stain viewed under polarized light shows abnormally expanded mantle dentin (MD) layer (note length of the white bar) in *Phospho1*^−/−^ vs. WT mice, where DE mineralization defects are focused. **(H, K)** von Kossa stain shows patchy osteomalacia (yellow arrows) characteristic of *Phospho1*^−/−^ mouse AB defects. **(L, M)** 2D micro-CT renderings with cellular cementum (CC) segmented (shown in yellow) reveal increased CC in *Phospho1*^−/−^ vs. WT mice by 3 mo. **(N)** Quantitative analysis confirms increased CC volume and similar mineral density in *Phospho1*^−/−^ vs. WT mice. Detailed analytical methods are included in the original publication. Micro-CT analysis was performed as described in Figure 1, and as described in Chavez et al., JBMR Plus 5(3): e10474, 2021, with the segmentation of cellular cementum from dentin accomplished using a median filter. Statistical analyses included independent samples *t*-tests where graphs show means ± SD and results are indicated by: *p < 0.05; **p < 0.01. **(F, H, I, and K)** are republished with permission from Zweifler et al. (29). The remaining images are expanded, previously unpublished analyses of samples presented in the above publication.

Dental development in *Phospho1*^−/−^ mice has been previously described (29, 43); however, in this study, we reanalyzed samples using high-resolution micro-CT in combination with previous histological observations to provide additional insights into the consequences of *Phospho1* deletion on dental development. Analyses were performed to parallel those reported for HPP mouse models described above (34, 35), to allow more direct comparisons. Overall, deletion of *Phospho1* leads to milder mineralization defects than global or conditional deletion of *Alpl* (Figures 2A–D). Compared to WT controls, *Phospho1*^−/−^ mice show enamel hypoplasia (Figure 2E), and a previous report indicated disturbed enamel ultrastructure and reduced mineralization in continuously erupting mouse incisors (44). Dentin volume increases in *Phospho1*^−/−^ vs. WT mice by 3 months, although a small but significant reduction in dentin mineral density is evident. Alveolar bone density is also negatively affected by the loss of PHOSPHO1.

Histology provides further insights into how the absence of PHOSPHO1 affects dentoalveolar tissues, and we focus on periodontal tissues to contrast important differences arising from loss-of-function of TNAP vs. PHOSPHO1. Unlike in HPP models, an apparently normal acellular cementum layer is present in *Phospho1*^−/−^ mice and PDL attachment remains intact (Figures 2F–I), corresponding to normal alveolar crest height (yellow dotted lines in Figures 2A vs. 2C). The outer dentin layer of *Phospho1*^−/−^ vs. WT mice shows altered collagen organization, indicative of the mineralization defect that is limited to mantle dentin (Figures 2G–J). Alveolar bone in *Phospho1*^−/−^ shows “patchy” osteomalacia (Figures 2H,K) that has been described for bone in other skeletal locations (45). Intriguingly, cellular cementum volume nearly doubles in *Phospho1*^−/−^ vs. WT mice by 3 months (Figures 2L–N).

The pattern of defects with loss-of-function of PHOSPHO1 closely match the developmental expression, as explored by mRNA *in situ* hybridization, and protein immunohistochemistry, immunogold labeling, and western blot (29, 43, 44). Expression was noted in ameloblasts, odontoblasts, alveolar bone osteoblasts, and cementoblasts associated with cellular cementum. MVs, wherein PHOSPHO1 functions, have been associated with mineralization by all these cells (46, 47). Strikingly, PHOSPHO1 expression was not identified in cementoblasts during acellular cementum formation, no acellular cementum defect was noted in mice lacking PHOSPHO1, and MVs have not been associated with acellular cementum (29, 46).

## PREDICTING DENTAL MANIFESTATIONS IN PATIENTS WITH PSEUDO-HPP FROM PHOSPHO1 MUTATIONS

The nomenclature of pseudo-HPP has sometimes been used to describe apparent cases of HPP wherein clinical presentation overlaps with manifestations of HPP, but where ALP levels are within the normal range (23, 24, 26, 27, 48–50). Many studies also report cases where clinical signs of HPP are present and ALP is low or normal, and no genetic variant in *ALPL* can be identified (51–55). By Sanger sequencing, up to 5% of suspected HPP cases resist the identification of a genetic factor (54–57). These cases are sometimes attributed to *ALPL* variants that are difficult to locate by sequencing (e.g., in regulatory regions of the gene). It should also be noted that a comprehensive differential diagnosis for low ALP will include other genetic conditions (e.g., osteogenesis imperfecta, forms of hypophosphatemic rickets, cleidocranial dysplasia, and Wilson’s disease), altered nutritional states (e.g., vitamin C, magnesium, or zinc deficiency, Celiac disease, malnutrition, or starvation), endocrine and mineral metabolism disorders (e.g., hypothyroidism, Cushing’s syndrome, and milk-alkali syndrome), and potential effects of medications (e.g., bisphosphonates, other antiresorptive drugs, and chronic corticosteroid treatment) (31, 58). Additionally, we propose that a subset of these individuals with low ALP that resist diagnosis could potentially carry unreported *PHOSPHO1* variants.

In a patient with suspected HPP that had no detectable *ALPL* variants, Taillandier et al. mapped an inherited heterozygous c. 95_97CCT deletion in *PHOSPHO1*, in addition to a heterozygous *COL1A2* mutation in a newborn with severe mineralization defects (57). *In vitro* functional tests using COS-1 fibroblast-like cells that transfected with a pCMV expression vector carrying the *PHOSPHO1* deletion did not indicate enzymatic loss-of-function using a modified PEA substrate. However, these tests may not recapitulate *in vivo* function (particularly with this complex genetic background), and long-term clinical manifestations in this patient carrying *PHOSPHO1* and *COL1A2* variants were not reported. Little work has been done defining the effects of mutation on PHOSPHO1 using functional assays, though mutation of key active site residues resulted in the loss of enzymatic activity (59). It is also possible that PHOSPHO1 loss-of-function could be compensated by additional regulators, including TNAP. Compensation and competition between mineralization regulators are complex, sometimes with age-dependent changes in compensatory mechanisms (11, 43, 60–63), and we propose that this is an aspect for further study in HPP and pseudo-HPP.

Skeletal manifestations resulting from loss-of-function of TNAP vs. PHOSPHO1 share significant overlap based on the mouse models (6, 11, 43). Consideration of the dentoalveolar effects from loss-of-function of TNAP vs. PHOSPHO1 provides a valuable opportunity to not only contrast the physiological functions of these phosphatases but may also provide clinical utility. While enamel, (mantle) dentin, and alveolar bone are affected similarly by loss-of-function of these phosphatases, there is one key difference that stands out from the accumulated preclinical data. PHOSPHO1 deficiency does not appear to negatively affect acellular cementum or periodontal function. This observation can guide a prediction of the dentoalveolar phenotype to be expected in individuals who may carry pathogenic *PHOSPHO1* variants. First, relatively mild enamel and dentin defects may exist in such an individual and could manifest as enamel hypoplasia and dentin abnormalities focused in the mantle dentin (e.g., altered appearance by histology, such as interglobular patterns). Both types of alterations might be subclinical in that they may be so mild as to not cause detectable pathology. Second, and most importantly, periodontal attachment would likely not be impaired by *PHOSPHO1* mutations. Acellular cementum appeared undiminished (even increased in thickness) in mice lacking *Phospho1*, PDL attachment was intact, and alveolar bone levels were unaltered (29). This paints a picture in contrast to HPP, where premature tooth loss from cementum and periodontal attachment defects represents the most consistent and common dental effect.

Such a hypothetical individual presenting mild enamel and/or dentin defects and normal periodontal function could potentially be diagnosed from the dental presentation with amelogenesis imperfecta (AI; OMIM# 301200, 204650, 104500, 612529, 204700, and many more), dentinogenesis imperfecta (DI) type II or III (OMIM# 125490, 125500), dentin dysplasia type II (DD; OMIM# 125420), while potential skeletal effects of a *PHOSPHO1* mutation (e.g., scoliosis, fractures, and osteomalacia) may suggest one of the many forms of osteogenesis imperfecta (OMIM# 166200, 166210, and several others). HPP would also be in the differential diagnosis, and normal or low ALP in combination with lack of detectable *ALPL* mutation(s) could be labeled as an unusual manifestation of HPP or pseudo-HPP. Based on the accumulated evidence presented in this perspective article, we encourage clinicians to consider *PHOSPHO1* as a genetic sequencing target in cases of suspected HPP where mutations in *ALPL* cannot be identified and include it in genetic testing panels for endocrine and mineral metabolism disorders, an approach increasingly gaining in popularity (57, 64, 65).

A dissenting opinion may be that *PHOSPHO1* loss-of-function variants have not been identified in humans because they are not compatible with life. For example, the types of mineralization defects that are tolerable in *Phospho1*^−/−^ mice may be more severe and embryonic lethal in humans, though harmless when heterozygous (an unusual scenario). PHOSPHO1 expression in testes may affect fertility and thus the heritability of mutations. This is an area deserving further study.

## CONCLUSION

In this perspective article, we compared and contrasted the loss-of-function effects of mineralization-associated phosphatases, TNAP and PHOSPHO1, on the dentoalveolar complex, based on preclinical and case report studies. To date and to our knowledge, *PHOSPHO1* loss-of-function variants have not been reported in humans. Based on the accumulated evidence, we propose that there may be a subset of cases of pseudo-HPP associated with *PHOSPHO1* mutations. These would be expected to have skeletal effects such as osteomalacia, and fractures, consistent with HPP, but featuring dental phenotypes with mild enamel and dentin effects and intact cementum, periodontal attachment, and no premature tooth loss. Further research is clearly needed to identify such individuals if they remain undiagnosed or misdiagnosed under current medical paradigms.

## Data Availability

The raw data supporting the conclusions of this article will be made available by the authors, without undue reservation.

## References

[1] Glimcher M. Bone: nature of the calcium phosphate crystals and cellular, structural, and physical chemical mechanisms in their formation. Rev Mineral Geochem. (2006) 64:223–82. doi:10.1515/9781501509421-009

[2] McNally EA, Schwarcz HP, Botton GA, Arsenault AL. A model for the ultrastructure of bone based on electron microscopy of ion-milled sections. PLoS ONE. (2012)7:e29258. doi: 10.1371/journal.pone.0029258 22272230 PMC3260135

[3] Anderson HC. Vesicles associated with calcification in the matrix of epiphyseal cartilage. J Cell Biol. (1969) 41:59–72. doi: 10.1083/jcb.41.1.59 5775794 PMC2107736

[4] Ali SY, Sajdera SW, Anderson HC. Isolation and characterization of calcifying matrix vesicles from epiphyseal cartilage. Proc Natl Acad Sci USA. (1970) 67:1513–20. doi: 10.1073/pnas.67.3.1513 5274475 PMC283384

[5] Bonucci E. Fine structure and histochemistry of “calcifying globules” in epiphyseal cartilage, Z Zellforsch Mikrosk Anat. (1970) 103:192–217. doi: 10.1007/BF00337312 5412827

[6] Millan JL. The role of phosphatases in the initiation of skeletal mineralization. Calcif Tissue Int. (2013) 93:299–306. doi: 10.1007/s00223-012-9672-8 23183786 PMC3594124

[7] Roberts SJ, Stewart AJ, Sadler PJ, Farquharson C. Human PHOSPHO1 exhibits high specific phosphoethanolamine and phosphocholine phosphatase activities. Biochem J. (2004) 382:59–65. doi:10.1042/BJ20040511 15175005 PMC1133915

[8] Ciancaglini P, Yadav MC, Simao AM, Narisawa S, Pizauro JM, Farquharson C, Kinetic analysis of substrate utilization by native and TNAP-, NPP1-, or PHOSPHO1-deficient matrix vesicles. J Bone Miner Res. (2010) 25:716–23. doi: 10.1359/jbmr.091023 19874193 PMC3153326

[9] Anderson HC, Hsu HH, Morris DC, Fedde KN, Whyte MP. Matrix vesicles in osteomalacic hypophosphatasia bone contain apatite-like mineral crystals. Am J Patho1. (1997) 151:1555–61. PMC1858375 9403706

[10] Anderson HC, Sipe JB, Hessle L, Dhanyamraju R, Atti E, Camacho NP, Impaired calcification around matrix vesicles of growth plate and bone in alkaline phosphatase-deficient mice. Am J Pathol. (2004) 164:841–7. doi: 10.1016/S0002-9440(10)63172-0 14982838 PMC1613274

[11] Yadav MC, Simao AM, Narisawa S, Huesa C, McKee MD, Farquharson C, Loss of skeletal mineralization by the simultaneous ablation of PHOSPHO1 and alkaline phosphatase function: a unified model of the mechanisms of initiation of skeletal calcification. J Bone Miner Res. (2011)26:286–97. doi: 10.1002/jbmr.195 20684022 PMC3179344

[12] Millan JL, Whyte MP. Alkaline phosphatase and hypophosphatasia. Calcif Tissue Int. (2016) 98:398–416. doi: 10.1007/s00223-015-0079-1 26590809 PMC4824800

[13] Whyte MP, Greenberg CR, Salman NJ, Bober MB, McAlister WH, Wenkert D, Enzyme-replacement therapy in life-threatening hypophosphatasia. N Engl J Med. (2012) 366:904–13. doi:10.1056/NEJMoa1106173 22397652

[14] Silver MM, Vilos GA, Milne KJ. Pulmonary hypoplasia in neonatal hypophosphatasia. Pediatr Pathol. (1988) 8:483–93. doi: 10.3109/15513818809022304 3227000

[15] Shohat M, Rimoin DL, Gruber HE, Lachman RS. Perinatal lethal hypophosphatasia; clinical, radiologic and morphologic findings. Pediatr Radiol. (1991) 21:421–7. doi: 10.1007/BF02026677 1749675

[16] Whyte MP, Leung E, Wilcox WR, Liese J, Argente J, Martos-Moreno G, Natural history of perinatal and infantile hypophosphatasia: a retrospective study. J Pediatr. (2019) 209:116–24.e114. doi: 10.1016/j.jpeds.2019.01.049 30979546

[17] Foster BL, Nociti FH Jr, Somerman MJ. The rachitic tooth. Endocr Rev. (2014) 35:1–34 doi:10.1210/er.2013-1009 23939820 PMC3895863

[18] Reibel A, Maniere MC, Clauss F, Droz D, Alembik Y, Mornet E, Orodental phenotype and genotype findings in all subtypes of hypophosphatasia. Orphanet J Rare Dis. (2009) 4:6. doi: 10.1186/1750-1172-4-6 19232125 PMC2654544

[19] Foster BL, Ramnitz MS, Gafni RI, Burke AB, Boyce AM, Lee JS, Rare bone diseases and their dental, oral, craniofacial manifestations, J Dent Res. (2014) 93:7S–19. doi:10.1177/0022034514529150 24700690 PMC4107543

[20] Narisawa S, Frohlander N, Millan JL. Inactivation of two mouse alkaline phosphatase genes and establishment of a model of infantile hypophosphatasia. Dev Dyn. (1997) 208:432–46. 9056646 10.1002/(SICI)1097-0177(199703)208:3<432::AID-AJA13>3.0.CO;2-1

[21] Fedde KN, Blair L, Silverstein J, Coburn SP, Ryan LM, Weinstein RS, Alkaline phosphatase knock-out mice recapitulate the metabolic and skeletal defects of infantile hypophosphatasia. J Bone Miner Res. (1999) 14:201–26. doi: 10.1359/jbmr.1999.14.12.2015 PMC3049802 10620060

[22] Waymire KG, Mahuren JD, Jaje JM, Guilarte TR, Coburn SP, MacGregor GR. Mice lacking tissue non-specific alkaline phosphatase die from seizures due to defective metabolism of vitamin B-6. Nat Genet. (1995) 11:45–51 doi: 10.1038/ng0995-45 7550313

[23] Wenkert D, McAlister WH, Mumm S, Whyte MP. c.1250A>G, p.N417S is a common American TNSALP mutation involved in all clinical forms of hypophosphatasia (HPP), including pseudo-HPP. J Bone Miner Res. (2008) 23:S242.

[24] Sarkar AK, Ghosh SK, Mitra P, Mandal S, Mukhopadhyay S, Mathew J. Pseudohypophosphatasia. Indian J Pediatr. (1997) 64:256–61. doi: 10.1007/BF02752460 10771846

[25] Fedde KN, Cole DE, Whyte MP. Pseudohypophosphatasia: aberrant localization and substrate specificity of alkaline phosphatase in cultured skin fibroblasts. Am J Hum Genet. (1990) 47:776–83. 2171330 PMC1683693

[26] Heaton BW, McClendon JL. Childhood pseudohypophosphatasia. Clinical and laboratory study of two cases. Tex Dent J. (1986) 103:4–8. 3464116

[27] Scriver CR, Cameron D. Pseudohypophosphatasia. N Engl J Med. (1969) 281:604–6. doi: 10.1056/NEJM196909112811107 4309618

[28] Whyte MP. Hypophosphatasia - aetiology, nosology, pathogenesis, diagnosis and treatment. Nat Rev Endocrinol. (2016) 12:233–46. doi: 10.1038/nrendo.2016.14 26893260

[29] Zweifler LE, Ao M, Yadav M, Kuss P, Narisawa S, Kolli TN, Role of PHOSPHO1 in periodontal development and function. J Dent Res. (2016) 95:742–51. doi:10.1177/0022034516640246 27016531 PMC4914864

[30] Foster BL, Nagatomo KJ, Tso HW, Tran AB, Nociti FH Jr, Tooth root dentin mineralization defects in a mouse model of hypophosphatasia. J Bone Miner Res. (2013) 28:271–82. doi: 10.1002/jbmr.1767 22991301 PMC3541444

[31] Bowden SA, Foster BL. Alkaline phosphatase replacement therapy for hypophosphatasia in development and practice. Adv Exp Med Biol. (2019) 1148:279–322. doi: 10.1007/978-981-13-7709-9_13 31482504

[32] Bowden SA, Foster BL. Profile of asfotase alfa in the treatment of hypophosphatasia: design, development, and place in therapy. Drug Des Devel Ther. (2018) 12:3147–61. doi:10.2147/DDDT.S154922 PMC6161731 30288020

[33] Foster BL, Sheen CR, Hatch NE, Liu J, Cory E, Narisawa S, Periodontal defects in the A116T knock-in murine model of odontohypophosphatasia. J Dent Res. (2015) 94:706–14. doi:10.1177/0022034515573273 25716980 PMC4502784

[34] Foster BL, Kuss P, Yadav MC, Kolli TN, Narisawa S, Lukashova L, Conditional alpl ablation phenocopies dental defects of hypophosphatasia. J Dent Res. (2017) 96:81–91. doi:10.1177/0022034516663633 27582029 PMC5347426

[35] Kramer K, Chavez MB, Tran AT, Farah F, Tan MH, Kolli TN, Dental defects in the primary dentition associated with hypophosphatasia from biallelic ALPL mutations. Bone. (2021) 143:115732. doi: 10.1016/j.bone.2020.115732 33160095 PMC7769999

[36] Gasque KC, Foster BL, Kuss P, Yadav MC, Liu J, Kiffer-Moreira T, Improvement of the skeletal and dental hypophosphatasia phenotype in Alpl−/− mice by administration of soluble (non-targeted) chimeric alkaline phosphatase. Bone. (2015) 72:137–47. doi: 10.1016/j.bone.2014.11.017 25433339 PMC4283789

[37] Yadav MC, de Oliveira RC, Foster BL, Fong H, Cory E, Narisawa S, Enzyme replacement prevents enamel defects in hypophosphatasia mice. J Bone Miner Res. (2012) 27:1722–34. doi:10.1002/jbmr.1619 22461224 PMC3395779

[38] McKee MD, Nakano Y, Masica DL, Gray JJ, Lemire I, Heft R, Enzyme replacement therapy prevents dental defects in a model of hypophosphatasia. J Dent Res. (2011) 90:470–6. doi:10.1177/0022034510393517 21212313 PMC3144124

[39] Millan JL, Narisawa S, Lemire I, Loisel TP, Boileau G, Leonard P, Enzyme replacement therapy for murine hypophosphatasia. J Bone Miner Res. (2008) 23:777–87. doi:10.1359/jbmr.071213 18086009 PMC2652241

[40] Zweifler LE, Patel MK, Nociti FH Jr, Wimer HF, Millan JL, Counter-regulatory phosphatases TNAP and NPP1 temporally regulate tooth root cementogenesis. Int J Oral Sci. (2015) 7:27–41. doi:10.1038/ijos.2014.62 25504209 PMC4817535

[41] Williams DK, Pinzon C, Huggins S, Pryor JH, Falck A, Herman F, Genetic engineering a large animal model of human hypophosphatasia in sheep. Sci Rep. (2018) 8:16945. doi:10.1038/s41598-018-35079-y 30446691 PMC6240114

[42] Hu JC, Plaetke R, Mornet E, Zhang C, Sun X, Thomas HF, Characterization of a family with dominant hypophosphatasia. Eur J Oral Sci. (2000) 108:189–94. doi: 10.1034/j.1600-0722.2000.108003189.x 10872988

[43] McKee MD, Yadav MC, Foster BL, Somerman MJ, Farquharson C, Millan JL. Compounded PHOSPHO1/ALPL deficiencies reduce dentin mineralization. J Dent Res. (2013) 92:721–7. doi:10.1177/0022034513490958 23694930 PMC3711567

[44] Pandya M, Rosene L, Farquharson C, Millan JL, Diekwisch TGH. Intravesicular Phosphatase PHOSPHO1 function in enamel mineralization and prism formation. Front Physiol. (2017) 8:805. doi: 10.3389/fphys.2017.00805 29089903 PMC5651051

[45] Boyde A, Staines KA, Javaheri B, Millan JL, Pitsillides AA, Farquharson C. A distinctive patchy osteomalacia characterises Phospho1-deficient mice. J Anat. (2017) 231:298–308. doi:10.1111/joa.12628 28737011 PMC5522900

[46] Takano Y, Sakai H, Baba O, Terashima T. Differential involvement of matrix vesicles during the initial and appositional mineralization processes in bone, dentin, and cementum. Bone. (2000) 26:333–9. doi: 10.1016/S8756-3282(00)00243-X 10719275

[47] Takano Y, Sakai H, Baba O, Sakamoto Y, Terashima T, Ohya K, Demonstration of putative Ca-binding domains in dentin matrix of rat incisors after daily injections of 1-hydroxyethylidene-1,1-bisphosphonate (HEBP). Eur J Oral Sci. (1998) 106(Suppl. 1):274–81. doi: 10.1111/j.1600-0722.1998.tb02187.x 9541237

[48] Whyte MP, Walkenhorst DA, Fedde KN, Henthorn PS, Hill CS. Hypophosphatasia: levels of bone alkaline phosphatase immunoreactivity in serum reflect disease severity. J Clin Endocrinol Metab. (1996) 81:2142–8. doi: 10.1210/jcem.81.6.8964842 8964842

[49] Whyte MP. Hypophosphatasia and the role of alkaline phosphatase in skeletal mineralization. Endocr Rev. (1994) 15:439–61. doi: 10.1210/er.15.4.439 7988481

[50] Cole DE, Salisbury SR, Stinson RA, Coburn SP, Ryan LM, Whyte MP. Increased serum pyridoxal-5’-phosphate in pseudohypophosphatasia. N Engl J Med. (1986) 314:992–3. doi:10.1056/NEJM198604103141515 3960066

[51] Mornet E, Taillandier A, Domingues C, Dufour A, Benaloun E, Lavaud N, Hypophosphatasia: a genetic-based nosology and new insights in genotype-phenotype correlation. Eur J Hum Genet. (2021) 29:289–99. doi: 10.1038/s41431-020-00732-6 32973344 PMC7868366

[52] Garda-Fontana C, Villa-Suarez JM, Andujar-Vera F, Gonzalez-Salvatierra S, Martinez-Navajas G, Real PJ, Epidemiological clinical and genetic study of hypophosphatasia in a spanish population: identification of two novel mutations in the alpl gene. Sci Rep. (2019) 9:9569. doi:10.1038/s41598-019-46004-2 31267001 PMC6606844

[53] Fauvert D, Brun-Heath I, Lia-Baldini AS, Bellazi L, Taillandier A, Serre JL, Mild forms of hypophosphatasia mostly result from dominant negative effect of severe alleles or from compound heterozygosity for severe and moderate alleles. BMC Med Genet. (2009) 10:51. doi: 10.1186/1471-2350-10-51 19500388 PMC2702372

[54] Hepp N, Frederiksen AL, Duno M, Praest Holm J, Rye Jorgensen N, Beck Jensen JE. Biochemical clinical and genetic characteristics in adults with persistent hypophosphatasaemia; data from an endocrinological outpatient clinic in Denmark Bone Rep. (2021) 15:101101. doi: 10.1016/j.bonr.2021.101101 34258332 PMC8256181

[55] Jandl NM, Schmidt T, Rolvien T, Sturznickel J, Chrysostomou K, von Vopelius E, Genotype-phenotype associations in 72 adults with suspected ALPL-associated hypophosphatasia. Calcif Tissue Int. (2021) 108:288–301 doi: 10.1007/s00223-020-00771-7 33191482 PMC7881968

[56] Mornet E Hypophosphatasia: the mutations in the tissue-nonspecific alkaline phosphatase gene. Hum Mutat. (2000) 15:309–15. doi: 10.1002/(SICI)1098-1004(200004)15:4<309∷AID-HUMU2>3.0.CO;2-C 10737975

[57] Taillandier A, Domingues C, De Cazanove C, Porquet-Bordes V, Monnot S, Kiffer-Moreira T, Molecular diagnosis of hypophosphatasia and differential diagnosis by targeted next generation sequencing. Mol Genet Metab. (2015) 116:215–20. doi: 10.1016/j.ymgme.2015.09.010 26432670 PMC5257278

[58] Schmidt T, Schmidt C, Amling M, Kramer J, Barvencik F. Prevalence of low alkaline phosphatase activity in laboratory assessment: Is hypophosphatasia an underdiagnosed disease? Orphanet J Rare Dis. (2021) 16:452. doi: 10.1186/s13023-021-02084-w 34711245 PMC8555173

[59] Roberts SJ, Stewart AJ, Schmid R, Blindauer CA, Bond SR, Sadler PJ, Probing the substrate specificities of human PHOSPHO1 and PHOSPHO2. Biochim Biophys Acta. (2005) 1752:73–82. doi: 10.1016/j.bbapap.2005.06.009 16054448

[60] Harmey D, Hessle L, Narisawa S, Johnson KA, Terkeltaub R, Millan JL. Concerted regulation of inorganic pyrophosphate and osteopontin by akp2, enpp1, and ank: an integrated model of the pathogenesis of mineralization disorders. Am J Pathol. (2004) 164:1199–209. doi: 10.1016/S0002-9440(10)63208-7 15039209 PMC1615351

[61] Harmey D, Johnson KA, Zelken J, Camacho NP, Hoylaerts MF, Noda M, Elevated skeletal osteopontin levels contribute to the hypophosphatasia phenotype in Akp2(−/−) mice. J Bone Miner Res. (2006) 21:1377–86. doi: 10.1359/jbmr.060619 16939396

[62] Murshed M, Harmey D, Millan JL, McKee MD, Karsenty G. Unique coexpression in osteoblasts of broadly expressed genes accounts for the spatial restriction of ECM mineralization to bone. Genes Dev. (2005) 19:1093–104. doi: 10.1101/gad.1276205 15833911 PMC1091743

[63] Ao M, Chavez MB, Chu EY, Hemstreet KC, Yin Y, Yadav MC, Overlapping functions of bone sialoprotein and pyrophosphate regulators in directing cementogenesis. Bone. (2017) 105:134–47. doi: 10.1016/j.bone.2017.08.027 28866368 PMC5730356

[64] Rush ET, Johnson B, Aradhya S, Beltran D, Bristow SL, Eisenbeis S, Molecular diagnoses of X-linked and other genetic hypophosphatemias: results from a sponsored genetic testing program. J Bone Miner Res. (2021). doi: 10.1002/jbmr.4454. [Epub ahead of print]. PMC9298723 34633109

[65] Rey T, Tarabeux J, Gerard B, Delbarre M, Le Bechec A, Stoetzel C, Protocol genoDENT: implementation of a new NGS panel for molecular diagnosis of genetic disorders with orodental involvement. Methods Mol Biol. (2019) 1922:407–52. doi: 10.1007/978-1-4939-9012-2_36 30838594

